# Anaemia and its determinants among reproductive age women (15–49 years) in the Gambia: a multi-level analysis of 2019–20 Gambian Demographic and Health Survey Data

**DOI:** 10.1186/s13690-022-00985-1

**Published:** 2022-11-09

**Authors:** Kegnie Shitu, Bewuketu Terefe

**Affiliations:** 1 Department of Health Education and Behavioral Science, Institute of Public Health, College of Medicine and Health Sciences, University of Gondar, Gondar, Ethiopia; 2 Department of Community Health Nursing, School of Nursing, College of Medicine and Health Sciences, University of Gondar, Gondar, Ethiopia

**Keywords:** Anemia, Determinants, Gambia, Multi-level Analysis, Reproductive age women

## Abstract

**Background:**

Anaemia is among the top list of the contemporarily public health burden in both developed and developing countries, by affecting mainly women's and children's health.

**Objective:**

This study aimed to identify the burden of anaemia and its individual and community level factors among women in The Gambia.

**Method:**

This study was based on an extensive national survey, Gambian Demographic and Health Survey. A total weighted sample of 5,858 reproductive-age women was included. Because of the hierarchical nature of the DHS data, a multi-level logistic regression model was applied to study individual and community-level factors that may influence anaemia. A 95% confidence interval and a *p*-value of less than 0.05 were used to declare statistical significance.

**Result:**

The overall prevalence of anemia was found 44.28% (95% CI 0.43, 0.46). Current users of contraceptives were (AOR = 0.66, 95% CI: (0.55- 0.79)) and currently pregnant (AOR = 1.44. 95% CI: (1.16, 1.81)) less likely and more likely to develop anaemia compared to their counterparts respectively. In addition to this, living in the region of Brikama (AOR = 0.69, 95% CI: (0.50–0.97)) less likely to be exposed to anemia. From community level factor, high distance to the health facilities (AOR = 1.23,95% CI 1.02–1.48) were associated with anemia.

**Conclusion:**

The study revealed that the burden of anaemia among reproductive age Gambian women was very high. Anaemia was affected by both individual and community levels of factors. Thus, the burden of anaemia could be significantly reduced if pregnant and contraceptive users' women were monitored and encouraged. Increasing the accessibility of health facilities, community mobilization, and awareness enhancement are also advisable.

## Introduction

Anaemia could hold the definition of reducing the number of red blood cells, haemoglobin and hematocrit below the acceptable range of any healthy person keeping constant other variables such as age, sex and race, however, under similar environmental situations [1]. Although anaemia affects all ages, it mainly affects children (42%) and pregnant and non-pregnant females particularly in low and middle-income countries than the general population (40%) [2, 3]. Age, sex and physiological status of individuals will assist clinicians to classify those individuals, whether not anaemic, middle, moderate or severe, according to their level of haemoglobin concentration in their blood [4]. Various studies indicate that anaemia increases people’s risk of other illnesses (heart failure and tuberculosis), disturbing sleep quality, financial and other social and psychological stress [5–7]. The physiological and physical alternations with higher nutritional requirements make adolescents among the higher risk group of anaemia [8]; this also make them the most risky and vulnerable group during their motherhood experience [9]. however, they are the most ignored groups of people and not sufficiently addressed by researchers and accessing of healthcare services, mainly in Africa [10]. According to the WHO in 2019 and 204 countries and territories, anemia affects about 29.9% of mothers of childbearing age and 39.9% of children under the age of five, affecting a total of about half billion reproductive age women [11, 12].

Africa is the most severely attacked continent of the rest world. The world health organization 2015 report indicated that about 10.8 and 9.7 million women are exposed to anaemia in Africa and the Pacific regions, respectively [13]. The prevalence of anaemia is increasing at an alarming rate in Africa among non-pregnant from 37.7% to 41.5% and from 38.9% to 48.7% for pregnant women with an overall all prevalence of 62.3% [14]. As anaemia affects billions of people worldwide and exposes them to various social, psychological and economic crises, governmental and non-governmental organizations worldwide are paying close attention as a public health concern [15–18]. According to the Gambian micronutrient survey of 2018 report, Gambia is experiencing double or tripled burdens of malnutrition exaggerated by multiple problems of poverty [19]. Several studies were conducted to identify the possible risk or associated factors of anaemia and other related malnutrition. Studies conducted in sub-Saharan Africa, Ethiopia, Lao, Nigeria and Yemen identified that place of residence, family size, poor sanitation, educational status, age, pregnancy complications, meal frequencies and diversity scores were associated with anemia [20–25]. Regarding to prevalence of anemia number of studies depicted different results across countries, 39%, 19.7% Ethiopia [21, 22], 39.2% Japan [24], 42% Ghana [26], 25% Yemen [25], 7.6% South Africa [27], 68% Gambia [28]. Another study in the Gambia among non-pregnant women also showed that the prevalence is about 41.4% for iron deficiency and 28.0% for iron deficiency anemia, respectively [29].

Reports showed that the prevalence of anaemia among reproductive Gambian women is about 57.5%, and its ever lowest prevalence of anaemia was 56.5% in 2011 [30]. However, the Gambia Demographic Health Survey (GDHS) 2019–20 shows the lower prevalence of anaemia among pregnant women was 44% [31]. Despite growing international attention to anaemia, the Gambian government has shown a weak policy implementation and attitude in this regard. Evidence for this, the 2018 report of joint assessment of national strategies concluded that nearly half of stakeholders are not satisfied with the implementation of Gambian government strategies [32] so that health policy and health systems of this country’s needs tremendous implementation efforts and evidence from researchers to avert it [33]. Though the Government of The Gambia has put several measures to address undernutrition, the prevalence of micronutrient deficiencies is still alarmingly high [34, 35]. Therefore, this study aimed to identify potential determinants of anaemia in Gambian women to help stakeholders and program implementers by providing accurate and timely findings.

## Methods

### Study setting

The Gambia is situated on the western coast of Africa, and it is long and narrow in shape. The country is bound on three sides by Senegal and on the west by the Atlantic Ocean. The Gambia is one of the smallest states in West Africa, with a land area of 10,689.28 km^2^. The population of the Gambia in 2013 is estimated to be 1.85 million with an estimated 50.41% of female population, and more than half of the population live in urban and semi-urban areas [36].

### Data source, population, and sampling procedure

The present study was based on the most recent Gambian Demographic and Health Survey (GDHS) data of 2019–20. A stratified two-stage cluster sampling technique was employed. In the first stage, 281 EAs were selected. In the second stage, an average of 25 households was selected per cluster/EA. We accessed the dataset used for the present study after registering and receiving an authentication letter from the Demographic and Health Survey (DHS) program at The DHS Program—Gambia: Standard DHS, 2010–20 Dataset. A total weighted sample of 5,858 reproductive-age women was included for this study [37].

### Variables of the study

The outcome variable of this study was anaemia among reproductive-age women. The variable was dichotomized into **0** = “Not anemic” and **1** = “anemic". The independent variables were classified into individual level (level 1) variables and community level (level 2) variables. Individual-level variables included age, religion, educational status, husband's/partner's age and education, family wealth index, current working status, family planning message exposure, knowledge of family planning methods, ever use of contraceptive, fertility preference, desire to have children, current pregnancy, and several children. Whereas, community variables involved variables directly taken with no aggregation (residence and contextual region), and variables obtained by aggregating individual variables into their respected community (community media exposure, community poverty, community women education, community distance to health facilities, community antenatal care service utilization rate, and community toilet facility). The aggregates were computed using the mean values of the proportions of women in each category of a given variable. Since the aggregate values of each variable do not follow a normal distribution curve, we categorized the aggregate values of a cluster into groups based on median values.

### Operational definitions

#### Community female education

This is the aggregate value of the educational levels of women based on the average of proportions of educational levels in the community. It was defined as low if the ratio of women with secondary education & above in the community was below the median and high if the value is higher than the media. The median value was 0.4286.

#### Community media exposure

This variable was derived from individual responses to radio or television exposure. It was defined as low if the proportion of women exposed to media in the community was 0–72% and high if the proportion was 73%–100%.

#### Community ANC utilization rate

This variable is also derived from the individual values for ANC utilization. It was defined as low if the proportion median of women who attended at least one ANC visit in the community was 0 – 50% and high if the proportion was between 51 –100%.

#### Community poverty

This variable is also derived from an individual household's wealth index with the same procedure. It was defined as high if the proportion of women from the two lowest wealth quintiles in a given community was 33.4%–100% and low if the proportion was 0–33.3%

#### Community distance to the health facility

The variable was aggregated from individual perceived distance to a health facility is a big problem**.** It was categorized as low if the proportion of women who perceived health facility distance as a big problem in the community was 0–21.43% and categorized as high if the proportion was between 21.44% and 100%.

#### Community toilet facility improvement

This variable was aggregated from individual toilet facilities like that of the above variables. It was classified as low if the value is below the median value of 0–34.78% and high if it is between 34.79%-100%

### Data processing, procedure and analysis

Data were extracted from individual records (IR) files, and further coding and transformations were done using statistical software, STATA version 14. The weighted samples were utilized for Analysis to adjust for unequal probability of selection and non-response in the original survey. Since the Gambian demographic and health survey (GDHS) data applied multi-stage stratified cluster sampling techniques, the data have a hierarchical structure. In this manner, single-level logistic regression is not recommended because classical multiple regression techniques treat the units of Analysis as independent observations. One consequence of failing to recognize hierarchical structures is that standard errors of regression coefficients will be underestimated, leading to an overstatement of statistical significance. In this point of view, an advanced statistical model that takes the hierarchy of the data into account is required to draw valid inferences and conclusions. Therefore, a multivariable multi-level binary logistic regression model was used to estimate the fixed and random effects of the factors associated with anaemia. Four models were constructed. The first model, also called an empty or null model, was fitted without including any explanatory variables. This model was specified to decompose the variance that existed between communities.

The null model is essential for understanding the community variations. We used it as a reference to estimate how much community factors could explain the observed variations in the intention to use contraceptives. Moreover, this model was used to justify using a multi-level statistical framework as it is a litmus paper on whether multi-level or traditional logistic regression should be used. It was assessed using the Log-Likelihood Ratio test (LLR), Median Odds Ratio (MOR), Intra-class Correlation Coefficient (ICC), and Proportional Change of Variance (PCV). The second model contained only individual-level factors. The third had only community-level characteristics. In comparison, the final (fourth) model included both individual and community-level factors. Moreover, the model comparison was made using model deviance, a model with the lowest deviance selected for reporting and interpretation results.

## Results

### Study population characteristics

The data on 5,636 reproductive age (15–49) women were included in this Analysis, including 281 clusters nested in the eight regions. The detailed descriptive statistics of the study participants are presented in Table 1. The respondents' mean (± SD) age was 28.3 years (± 9.4 years), and around 40.1% of women were found under the range of 15–24 years. Nearly two-thirds (64.36%) of participants were married or living with a partner. About (42.72%) of the women had a secondary level of educational status, and most of them (96.92%) were followers of the Islamic religion. Regarding occupational status, wealth index, anaemia status, and ethicality group, 40.28% had no work, nearly a quarter of them (25.1%) are the richest class, 32.90% were from Mandinka/Jahanka ethnicity, and about 44.28% of participants had experienced anaemia. Only 45.12% of them had taken iron folate during their pregnancies, and more than half (58.06) had never tested for HIV status. Regarding the source of drinking water supply, around 93.95% of them had drunk unimproved water, and nearly half of them (49.24%) had scored an average body mass index (Table 1).

**Table 1:** Sociodemographic and other health-related characteristics of study participants included in the analysis: Gambian demographic and health survey, 2019–20 (*n* = 5,858)

Variables	Anaemia status		Total, n (%)
	Not anaemic,	Anaemic,	
Age			
15–24	1,352.60	1,020.77	2,373.37(40.51)
25–35	1,176.58	915.68	2,092.27(35.72)
36–49	734.91	657.59	1,392.51(23.77)
Religion			
Muslim	3,156.23	2,521.36	5,677.58(96.92)
Christian	104.61	72.68	177.28(3.03)
Others	3.27	0	3.27(0.06)
The highest educational level of mothers			
No-formal education	988.48	1,034.08	2,022.56(34.53)
Primary	507.84	435.96	943.81(16.11)
Secondary	1,527.81	976.67	2,504.48(42.75)
Higher	239.97	147.33	387.29(6.61)
Marital status of mothers			
Single	1,272.38	815.31	2,087.69(35.64)
Married	1,991.72	1,778.73	3,770.45(64.36)
Ethnicity			
Mandinka/jahanka	1,054.51	872.63	1,927.15(32.90)
Wollof	415.23	327.81	743.03(12.68)
Jola/karoninka	443.86	210.94	654.80(11.18)
Fula/tukulur/lorobo	550.03	525.62	1,075.65(18.36)
Serere	145.07	80.85	225.93 (3.86)
Sarahule	229.41	234.13	463.53(7.91)
creole/aku /marabout	18.13	9.65	27.77(0.47)
Ninjago	35.86	30.42	66.28 (1.13)
Bambara	38.51	36.37	74.88(1.28)
Non-Gambian	303.84	251.92	555.76(9.49)
Other	29.65	13.71	43.36(0.74)
Occupation status of participants			
No working	1,338.38	1,021.21	2,359.58(40.28)
Farmer	418.99	550.47	969.47(16.55)
Salaried worker	213.63	115.99	329.63(5.63)
Sales and trades	1,128.09	816.42	1,944.51(33.19)
Others	164.99	89.95	254.94 (4.35)
Wealth Index			
Poorest	428.09	547.95	976.05(16.66)
Poorer	507.22	503.58	1,010.81(17.25)
Middle	656.89	514.74	1,171.63(20.00)
Richer	698.32	530.93	1,229.25(20.98)
Richest	973.56	496.84	1,470.41(25.10)
Body mass index^a^			
< 18.5	299.85	262.51	562.36(14.22)
18.5–24.9	1,085.91	861.01	1,946.93(49.24)
25–29.9	516.90	359.71	876.61(22.17)
= > 30	369.85	198.32	568.17(14.37)
Current use of contraceptive			
Yes	468.14	268.15	736.29(12.57)
No	2,795.96	2,325.89	5,121.85(87.43)
Iron folate intake during pregnancy			
Yes	1,420.75	1,222.26	2,643.01(45.12)
No/don’t know	1,843.35	1,371.78	3,215.14(54.88)
Children ever born/Gravidity			
0	1,218.51	869.33	2,087.84(35.64)
1–3	1,076.94	817.23	1,894.17(32.33)
> = 4	968.65	907.48	1,876.14(32.03)
Children born in the last three years			
0	2,154.83	1,612.31	3,767.14(64.31)
1	954.29	834.04	1,788.34(30.53)
1 +	154.97	147.68	302.66(5.17)
Currently breastfeeding			
Yes	729.18	634.31	1,363.48(23.28)
No	2,534.92	1,959.74	4,494.66(76.72)
Currently pregnant			
Yes	199.19	241.51	440.70(7.52)
No	3,064.91	2,352.54	5,417.44(92.48)
Ever tested for HIV			
Yes	1,373.49	1,083.53	2,457.02(41.94)
No	1,890.61	1,510.52	3,401.13(58.06)
Source of drinking water			
Improved water	2,941.07	2,359.87	5,300.94 (93.95)
Unimproved water	198.09	143.51	341.60 (6.05)
Type of toilet facility			
Unimproved	808.66	867.12	1,675.77(29.70)
Improved	2,330.49	1,636.26	3,966.76(70.30)
Anemia status			
Severe anaemia	0	70.12	70.12 (1.20)
Moderate anemia	0	1,012.55	1,012.55(17.28)
Mild anemia	0	1,511.37	1,511.37(25.80)
No anaemia	3,264.10	0	3,264.10 (55.72)
Ever use of ANC			
Never	1,814.793	1,355.377	3,170.17 (54.12)
Yes	1,449.311	1,238.668	2,687.978 (45.88)

^a^ Body mass index was not measured for lactating and non-pregnant mothers

### Community level factors characteristics

The majority of participants lived in an urban area (72.95%), and (43.72%) of participants belonged to the region of Brikama. In similar way participants in low community media exposure (41.5%), low community women education (34.47%), low community poverty (63.59%), low community antenatal care utilization rate (63.04%), low community distance to health facilities (50.47%), and low community toilet facility 60.07% of the participants were observed respectively (Table 2).

**Table 2:** Community-level variables descriptive result of reproductive age women Gambian demographic and health survey, 2019–20(*n* = 5,858)

Variables	Anaemia n (%)		Total, n (%)
	No	Yes	
Residence			
Urban	2,568.09	1,705.31	4,273.39(72.95)
Rural	696.016	888.73	1,584.75(27.05)
Region			
Banjul	47.04	33.91	80.95(1.38)
Kanifing	780.27	524.42	1,304.69(22.27)
Brikama	1,571.52	989.74	2,561.26(43.72)
Mansakonko	107.65	120.63	228.28(3.90)
Kerewan	254.61	298.59	553.21(9.44)
Kuntaur	96.21	159.31	255.52(4.36)
Janjanbureh	134.98	156.62	291.61(4.98)
Basse	271.81	310.82	582.63(9.95)
Community media exposure			
Low	1,227.98	1,200.25	2,428.23 (41.45)
High	2,036.12	1,393.79	3,429.92(58.55)
Community level women education			
Low	948.734018	1,070.553	2,019.287(34.47)
High	2,315.37	1,523.491	3,838.861 (65.53)
Community-level poverty			
Low	2,233.48	1,491.61	3,725.09(63.59)
High	1,030.62	1,102.44	2,133.05(36.41)
Community ANC utilization rate			
Low	2,132.97	1,559.94	3,692.91(63.04)
High	1,131.13	1,034.11	2,165.24(36.96)
Community distance from health institutions			
Low	1,780.919	1,175.825	2,956.744(50.47)
High	1,483.185	1,418.2194	2,901.404(49.53)
Community-level toilet facility			
Low	2,177.12	1,458.78	3,635.89(62.07)
High	1,086.98	1,135.26	2,222.25(37.93)

### Factors associated with anemia

In the multi-level multivariable Analysis, current use of contraceptives, currently pregnant women, region and community distance to health facilities were significant factors associated with participant's anaemia status. Current users of contraceptives of participants were 0.34 times less likely to be experienced anaemia compared to current nonusers of contraceptives (AOR = 0.66, 95% CI: (0.55- 0.79)). Currently, pregnant participants have 1.44 times more likelihood of exposure to anaemia (AOR = 1.44. 95% CI: (1.16, 1.81)) than women who are not currently pregnant. In addition to this, women living in the region of Brikama have (AOR = 0.69, 95% CI: (0.50–0.97)) less likely to be exposed to anaemia. Regarding community-level variables, Women from a community having a high distance to the health facilities were (AOR = 1.23,95% CI 1.02–1.48)) times more likely to be experienced anaemia compared to participants residing in communities with low distance to health facilities (Table 3).

**Table 3:** Individual and community-level factors associated with anaemia among reproductive-age women in Gambia (*n* = 5,858)

Independent variables	Null model	Model I	Model III	Model IV
		AOR [95% CI]	AOR [95% CI]	AOR [95% CI]
Age of the respondent				
15–24	-	1	-	1
25–35	-	1.02(0.86, 1.21)	-	1.03(0.87–1.22)
36–49	-	1.11 (0.86, 1.42)	-	1.13(0.87–1.46)
Education				
No formal education	-	1	-	1
Primary	-	0.86(0.73–1.02)	-	0.87(0.74–1.04)
Secondary	-	0.85(0.73–0.99)	-	0.89(0.76–1.05)
Higher	-	1.07(0.76–1.49)	-	1.09(0.78–1.53)
Marital status				
Single	-	1	-	1
Married	-	1.06(0.89–1.25)	-	1.04(0.87–1.23)
Occupational status				
No working	-	1	-	1
Farmer		1.12(1.01–1.41)		1.14(0.96–1.35)
Salaried worker		0.79(0.56–1.10)		0.79(0.57–1.11)
Sales and trades		0.92(0.79–1.06)		0.94(0.81–1.09)
Others	-	0.71(0.51–0.98)	-	0.74(0.54–1.04)
Wealth index				
Poorest	-	1	-	1
Poorer		0.89(0.74–1.06)		0.98(0.81–1.18)
Middle		0.77(0.63–0.94)		0.93(0.74–1.17)
Richer		0.72(0.57–0.90)		0.91(0.69–1.20)
Richest	-	0.60(0.47–0.77)	-	0.79(0.59–1.08)
Current use of contraceptive				
Yes	-	0.65(0.55–78)	-	0.66(0.55–0.79)
No	-	1	-	1
Iron folate intake during pregnancy				
Yes	-	1.23(0.78–1.94)	-	1.21(0.77–1.91)
No	-	1	-	1
Total children ever born Conti*		1.03(0.99–1.07)		1.03(0.99–1.08)
Births in the last three years Conti*		1.14(0.97–1.35)		1.15(0.98–1.36)
Currently breastfeeding	-		-	
Yes		0.96(0.78–1.17)		0.96(0.79–1.16)
No	-	1	-	1
Currently pregnant				
Yes	-	1.45(1.16–1.81)	-	1.44(1.16–1.81)
No	-	1	-	1
Ever tested for HIV	-		-	
Yes	-	0.96(0.81–1.09)	-	0.95(0.83–1.08)
No		1		1
Source of drinking water				
Unimproved	-	1	-	1
Improved	-	1.12(0.85–1.48)	-	1.10(0.83–1.46)
Type of toilet facility				
Unimproved	-	1	-	1
Improved	-	0.94(0.81–1.06)	-	0.94(0.81–1.09)
Ever use of ANC				
Yes	-	0.74(0.47–1.18)	-	0.76(0.47–1.21)
Never	-	1	-	1
Type of residence		-		
Urban	-	-	1	1
Rural	-	-	1.96(1.65–2.33)	1.17(0.83–1.64)
Region		-		
Banjul	-	-	1	1
Kanifing	-	-	0.92(0.66–1.28)	0.87(0.62–1.20)
Brikama	-	-	0.85(0.62–1.18)	0.69(0.50–0.97)
Mansakonko		-	1.58(1.09–2.31)	0.87(0.56–1.35)
Korean	-	-	1.65(1.15–2.35)	1.04(0.69–1.57)
Kuntaur	-	-	2.30(1.59–3.33)	1.11(0.69–1.77)
Janjanbureh			1.57(1.09–2.26)	0.81(0.51–1.29)
Basse	-	-	1.59(1.13–2.26)	0.88(0.58–1.35)
Community media exposure				
Low	-	-	1	1
High	-	-	0.69(0.58–0.84)	1.01(0.83–1.23)
Community-women education				
Low	-	-	1	1
High	-	-	0.55(0.46–0.65)	0.91(0.71–1.16)
Community-level poverty				
Low	-	-	1	1
High	-	-	1.80(1.51–2.15)	0.97(0.70–1.34)
Community ANC utilization rate				
Low	-	-	1	1
High	-	-	1.53(1.28–1.84)	1.07(0.88–1.30)
Community to Health Facility distance problem				
High	-	-	1.27(1.07–1.52)	1.23(1.02–1.48)
Low	-	-	1	1
Community-level toilet facility				
Low	-	-	1	1
High	-	-	1.57(1.31–1.88)	1.02(0.81–1.29)
Random parameters and model comparison				
Community-level variance	0.42(0.060)	0.278(0.049)	0.24(0.043)	0.24(0.449)
ICC (%)	11.2	7.8	6.7	6.8
MOR (95% CI)	1.84 (1.70, 202)	1.62(150, 1.80)	1.60(1.46, 1.74)	1.58(1.46, 1.73)
PCV (%)	Reference	36.60	41.46	43.90
LR	-3985.76	-3774.81	-3945.98	-3762.66
DIC (-2LLR)	7,971.52	7,549.62	7,891.96	7,525.32

Conti* Continuous variable

### Prevalence of anemia

Estimated participants of 2,594 (44.28%, 95% CI 0.43, 0.46) of reproductive age women have experienced anaemia.

## Discussion

Since anaemia among women is a significant public health concern in low and middle-income countries [38], this study aimed to assess the burden of anaemia and its associated factors among reproductive age Gambian women. The proportion of participants exposed to anaemia was 44.28 (0.43–0.46). This prevalence is less observed than studies conducted in Gambia [28, 29]. It is almost in agreement with a study conducted in Tanzania and Ghana [39]; nevertheless, this figure is higher than studies investigated in Ethiopia 22.7% 22.1% [40, 41], Rwanda [42]. The possible justification for why this study's empirical findings are higher than the one mentioned above might be due to countries profiles of anaemia and other communicable diseases, participants' attitudes, knowledge, and educational backgrounds towards anaemia. On the other hand, this study found a significantly lower prevalence of anaemia, which might minimize confounding factors both on the individuals and community-level factors that could have positive or negative implications on anaemia status. The majority of those listed studies concluded the prevalence of anaemia based upon individual factors with minimal sample size, study settings and without consideration of community level factors that might have effect on anaemia.

The multi-level logistic regression analysis depicted those women who have current contraceptives have less likely to be anaemic patients than current contraceptive nonusers. This finding is supported by literature investigated in Ethiopia [41], Rwanda [42], 24 Sub-Saharan Africa study [43]. The DHS data of 12 developing countries was conducted to determine the existence and degree of contraceptive benefits other than prevention of unintended pregnancy found a reduction rate of 32% to 44% odds of anaemia exposure among contraceptive users [44]. This could be explained by modern contraceptives having a positive protective effect on menstrual bleeding, pregnancy, birth-related haemorrhages, and iron supplementation to prevent anaemia other than prevention of pregnancy [45]. The observed gain in haemoglobin could be due to the considerable reduction in cyclic blood loss frequently documented among contraceptive uses [46]. Further, the placebo iron pills provided with many contraceptive brands can also reduce anaemia.

The study also found that participants who are currently pregnant have a more advanced probability of developing anaemia than those who did not have a current pregnancy status. Indeed, this is a physiological fact supported by the international resarchers and similar to the study done in Ethiopia [47, 48]. During pregnancy, some physiological systems begin to work unusually because of the growth of the fetus, the placenta, a more considerable amount of blood circulation in the pregnant mother and depletion of haemoglobin; these situations might expose her to higher demand of iron-folic acid nutrients. However, due to many factors, most pregnant mothers in developing countries could not start their iron folate supplementation at the right time [49]. This physiological change also will contribute to the increment of plasma and reduction of blood viscosity [50]. Other several studies also in agreement the scenario of anaemia to pregnancy physiological relationship and infections could lead pregnant mothers to iron deficiency is thought to be caused by a combination of factors such as previously decreased iron supply, the iron requirements of growing fetus and expansion of maternal plasma volume and infections [28, 51].

Study participants residing in the region of Brikama have a less likely chance of developing anaemia than their counterparts of the Banjul region. Although differences across regions did not well investigated in the Gambia, studies from similar developing countries found a regional variation of anemia [41, 42]. This could be demonstrated by differences in anemia risk in the spread of communicable diseases associated to regional geographic conditions, food supply, availability of a variety of foods, accessibility, and use of healthcare facilities. [20]. Household dietary diversity scores among regions were assessed in 2020, and Brikama had showed only 2% of food groups of 0–2; however, this figure was about 12% in Banjul. Furthermore, food consumption by region was also assessed, and Brikama has shown 9.9% poorer and 86.5% acceptable food consumption, whereas Banjul region scored 11.8% poor and 76.5% acceptable food consumption, respectively [52]. This might have its contribution by increasing the risk of anaemia.

Those participants living with a high range of distance to the health facilities have a higher tendency to be exposed to anaemia than those living with a limited or low distance from the health facilities. This result agrees with a qualitative study conducted in the Gambia that prolonged transportation decreases their health-seeking behavior [53]. For instance, a study conducted in five east African countries showed that distance perceived as a big problem was one factor for the burden of anemia [54].

## Conclusion

This study revealed that the overall burden of anaemia among reproductive age Gambian women is very high. Anemia was statistically significantly correlated with current contraceptive use, current pregnancy, state, area, and community distance to health facilities. Therefore, the risk of anaemia could be significantly reduced if pregnant women are monitored by health professionals, community health workers, others governmental and non-governmental stakeholders and given iron supplementation; it will also be better if women use contraceptives. A policy on the use of contraceptives has been developed by the Gambian government. It will be preferable if the government puts the policy into effect in accordance with the current and other fresh initiatives while taking into account contextual elements. In light of the community-level factors, the health sector could extend access to health facilities in all parts of the country to save mothers who become ill and suffer from malnutrition due to distance from health facilities. Community mobilization is also crucial to increase community awareness.

### Study implications for policy and practice

The implication of this study was providing a nationwide preliminary information for those who are engaging in the women and children health intervention by identifying both individual and community level potential factors of anaemia in Gambian women to help stakeholders and program implementers by providing accurate and timely findings.

## Data Availability

Data for this study were obtained from the DHS Program through https://www.dhsprogram.com/
